# Milk and Milk-Contracts
*Previous articles appeared on May 3, 10, 17, and 24.


**Published:** 1913-05-31

**Authors:** Everitt E. Norton

**Affiliations:** Medical Superintendent of Isleworth Infirmary.


### MILK AND MILK-CONTRACTS.*

By EVERITT E. NORTON, M.D., D.P.H., etc., Medical Superintendent of Isleworth Infirmary.

### V.—The Carriage of Milk.

There are three factors of especial importance with regard to the conveyance of milk from the farm to the consumer in an institution—namely, the railway churn, the time occupied in transit, and the *temperature of the milk* during that time.

There is no longer a possibility of supplying any appreciable proportion of the milk required for such a population as that of London from the immediate vicinity. Nor is there any likelihood of change either of the districts from which milk is supplied or in the speed of railway transport. I understand that it is estimated that in 1907 some 190,000 cows were required to produce the milk supply of London, whilst less than 4,000 milch-cows were kept in the county of London. London will continue to depend on rail-borne milk, and several hours must be occupied upon the journey. It is unfortunate, but apparently inevitable, that it should require to be stipulated, as it is in one milk contract (in many ways an excellent one) which I have seen, that not more than eighteen hours shall elapse between the time of milking and that of delivery, and in another that the milk shall "not be more than twenty-four hours old when delivered"!

There is no excuse whatever for the use of railway churns so notoriously defective in construction as those I have described. Churns of improved type are made for which it is claimed that the milk is protected from pollution by dust and rain, and which certainly have many advantages in details of lid fitting; these cannot even be said to be relatively costly; a variety of patterns is on the market, and a careful selection should be made. "Ventilating" holes are objectionable and are quite unnecessary; they are generally to be seen upon the ornamental brass-mounted dairyman's delivery churn, and occasionally upon the railway churn. Some exposure of the churn to dust is unavoidable, but with one properly covered this becomes a less momentous risk. It is noticeable that new churns of the old pattern, with the highly unsuitable form of cover, are seldom to be seen at railway stations and elsewhere. This fact leads me to hope, as also does perusal of the price-lists of makers of utensils for the milk-trade, that an increasing use (stimulated by the Board of Agriculture) is being, and will be, made of churns of improved construction.

Having regard, then, to the fact that London milk must unavoidably be travelled milk, and that a long time must be spent upon the journey, the one all-important point remaining for consideration is that of its temperature during that time.

It is certain that there is no condition so favourable to the development of a high bacterial content in milk as the combination of conveyance by rail and warmth. Delépine says that "the degree of noxiousness acquired through infection is proportional to the length of time the milk has been kept, and the temperature which it has been exposed to, before it reaches the consumer." All observers are agreed in the conclusion that, of all attainable improvements, the one which would be of pre-eminent value is that all milk should be promptly and effectively cooled at the farm, and should be maintained at a comparatively low degree of temperature until it is delivered to the consumer. This special requirement for the transport of milk has received far wider recognition both in America and upon the Continent than in this country. In most cities in America there is an ordinance, reasonably well enforced, prohibiting the sale of milk found to be at a higher temperature than 50° F. (Eastwood). Even here some advance has been made, and milk is, with increasing frequency, subjected to cooling at farms. This cooling is only too often ineffective, being carried only to about the temperature of the water from any supply which may be available and being less in warm weather when the need for it is greater; thus (as Dr. Ralph Vincent says) "the milk seldom falls to a temperature as low as 60° F., except possibly in cold weather, while the precautions taken to keep the milk cool after this preliminary treatment are conspicuous by their absence."

Milk as it leaves the cow has a temperature of nearly 100° F. Acidity increases rapidly at any temperature approaching 65°, but at 40° F. bacterial development is practically at a standstill.

Dr. Arthur Eastwood says that in England some of the main obstacles to the delivery of chilled milk are: (i) the cost of ice; (ii) the absence of effective demand; (iii) the objection of the railway companies to provide suitable vans; and (iv) the difficulty of obtaining ice at the farms. "At present" (he says) "(ii) seems to be the most serious of these difficulties. If the better-class consumer, who ought to know the advantages of properly cooled milk in hot weather, and can well afford to pay a little extra for it, would insist on demanding it, the problem would at once become a suitable matter for practical discussion."

The position in this country with regard to the collection, storage, and usage of ice is very different from that in America, where higher summer temperatures prevail, the use of ice for many purposes is customary, and milk is conveyed over even greater distances. To set up and use apparatus such as would enable our farmers to rapidly cool milk, even in hot weather, and to transmit chilled milk to the consumer, is not only troublesome but expensive, and might be, in the case of small isolated farms, so expensive that the cost would imperil profit, and so be prohibitive. Of the relative high value of chilled, as compared with unchilled, milk to the London consumer there can be no question, and because good milk may cost more than bad it is not necessarily dear at the price. Means of cooling *must* be provided, and the time cannot be far distant when, no matter what may be the cost and on whom the cost may fall, the influence of authorities

* Previous articles appeared on May 3, 10, 17, and 24.

and of public opinion will cause such means to be adopted and to be adopted universally.

There are quite practicable methods whereby railway churns may be adapted to the purpose of protecting cooled milk from rise of temperature in transit. The principle of the Thermos and similar flasks and bottles does not seem to have been yet applied for this purpose, but churns with double walls and lids, having an intervening air-space, are in use, and have proved to be of value: such churns are more expensive, and their capacity is reduced in proportion to their bulk. Again, churns are advertised and used, both here and abroad, which have ice-chambers either in the bottom or attached to the lid; the latter is the preferable arrangement, and is (when ice is available, and the difficulty lies in securing a cheap and sufficient supply of ice) an excellent one.

It seems hardly necessary to remark that churns of milk should not be left standing at railway stations, and should be protected, both there and during conveyance thereto and therefrom, not only from dust and rain, but from exposure to sunshine.

During the greater part of the time occupied in sending it to London the milk is in a railway van. Here, again, much remains to be done. It is not sufficiently recognised that trucks open at the sides, dusty and dirty, and used for purposes other than those of milk-traffic, are utterly unsuitable for that traffic. If the vans used were specially constructed insulated or refrigerator vans, such as are used for various food-trade purposes in America, and were used for the conveyance of milk and milk churns only, the cooled milk placed in them would need but little further protection. Unfortunately, such conditions are far from being yet generally realised, and the much-needed improvements will only be effected when pressure has been brought to bear upon the railway companies by the milk-trade itself. The consumer can only bring influence to bear though the contractor and by assisting in the creation of a demand; the milk-trade must see that the railway companies bear their share of the burden of reform.

With the delivery of the milk to the purchasers or their representative the responsibility of the contractor ends, and, for my present purpose, I am concerned only with the question of those further dealings with it which are consequent upon the imperfections which it may have, or may be suspected of having, acquired before delivery.

Milk delivered to institutions is very seldom efficiently tested; it is very rare for any test whatever to be applied, except a perfunctory one intended to estimate the quantity of milk-fat. A skilled examination may be occasionally made in some instances, but regular observation is not kept; in most cases an analyst is never employed.

Only too frequently comfort is weakly derived from the fact that inspectors under the Food and Drugs Act are empowered to take samples, and may sometimes do so; the results of a public analyst's examination are seldom made known to the purchasers, and his work is practically valueless as a test of the general care and efficiency with which the contract is being carried out. Estimations such as that of acidity, as a test of staleness and bacterial development, are never systematically made, nor is any bacteriological examination performed.

Straining of the milk on delivery is of very doubtful utility, and should be unnecessary. Milk obtained and delivered under satisfactory conditions, and which has been strained at the farm, should contain nothing capable of being removed by further straining. Further, it is of comparatively little use to strain from milk after delivery particles from which already, during many hours' soaking, pollution has been proceeding.

So great confidence has come to be placed in the processes of pasteurisation and sterilisation that to question their utility or advisability is to attack a position strongly supported by public opinion. It is impossible to here discuss the question at any length. It must suffice to say, again, that the practice of heating milk has arisen only since defects and dangers have been recognised in the sources and in the methods of our supplies. Our aim should surely be to *prevent* contamination rather than to attempt to reduce its evil effects.

I do not imagine that anyone using, for immediate consumption, milk obtained from his own healthy cow would pasteurise it before use, whether for infant-feeding or for any other purpose.

It has already been remarked that (there being at present in this country no restriction upon the sale of milk which has been pasteurised) it is not possible to say to what extent heating is practised in the trade. In all the large cities in America visited by him, Dr. Eastwood found the custom of pasteurising milk to be common, particularly amongst the larger dealers. In Minneapolis he was told that about 40 per cent. of the city's supply is pasteurised; in New York the percentage is said to be 25, and in Philadelphia 75. Boston, in 1906, received 368,849 quarts of milk daily, and of these 123,250 quarts were pasteurised.

Heating of milk is practised for the purpose of:—

(i) Delaying the bacterial development which causes souring.

(ii) Destroying pathogenic organisms, if any, contained in the milk.

Dr. Eastwood says: "Dealers pasteurise their milk in order to make it keep longer. This is the main, and generally the only, reason."

The custom of subjecting milk to treatment by heat in order to arrest the process of "turning" is a confession of weakness, and obviously involves a condemnation of the milk which needs such treatment.

The purchaser of milk should certainly not find it necessary to attempt to render it fit for use after it has become stale through age or through having been improperly kept. Moreover, it must not be forgotten that milk in which lactic acid change has been arrested by means of heat may, if further stored, become the happy hunting-ground of organisms far more harmful than those destroyed.

Milk which has been boiled undergoes a putrefactive decomposition, in which no development of lactic acid occurs.

